# Notes and News

**Published:** 1888-09-01

**Authors:** 


### Notes and News.

Collected and Communicated.

Mr. Henry Tonks, F.R.C.S., L.R.C.P., formerly of the London Hospital, has been appointed Senior Resident Medical Officer to the Royal Free Hospital, Gray's Inn Road.

Subscriptions on behalf of the Children's Fresh Air Mission, which last year sent 1,762 children living in Holborn, Clerkenwell, and surrounding districts, for two or three weeks in the country, are invited by Mr. W. Hazell, 6, Kirby Street, Hatton Garden.

A temperance procession will be formed on Sunday, September 9th, and march to St. Paul's Church, Peartree Street, Goswell Road, where a sermon will be preached by the Rev. A. S. Herring, M.A., on behalf of the Royal Hospital for Diseases of the Chest.

The Hospital Saturday collection in London this year amounts to £5,000, and exceeds that of last year by £500. When the workshop collections have been all paid in, it is expected that there will be a sum exceeding £10,000 available for distribution.

A garden party, under the patronage of Mr. S. Montagu, M.P., of Old Broad Street, will be given at the Essex County Cricket Ground on Wednesday, in aid of the funds of the Shadwell Hospital for Children, Victoria Park Hospital and the East London General Pension Society.

The town of Osnabruck has been visited by an extraordinary epidemic of ophthalmia militaris, which is particularly dreaded as leading, under certain conditions, to total blindness. A report received from the afflicted town, states there is hardly a house but has one of its inmates stricken.

Mr. Howard J. Collins, late secretary of the Hospitals Association, has been appointed secretary to the Norfolk and Norwich Hospital. The Hospitals Association, in recognition of Mr. Collins' unvarying courtesy and valuable services, are about to present him with an honorarium. The Norfolk and Norwich Hospital has secured in its new secretary a courteous, conscientious, and devoted officer.

"Hospitals, and not Asylums, for the Insane" is the keynote of a correspondence in the *Times* on the very important question of the treatment of lunatics.

It is proposed to take 250 of the most helpless of the inmates of the infirm wards of Lambeth Workhouse for a day in the country. For this purpose Mr. Howlett, chairman of the board, will gladly receive donations at 329, Wandsworth Road, S.W.

At a meeting of the Worcester Infirmary Committee, on August 20th, Mr. G. J. R. Fletcher, of Dulwich, was appointed house surgeon, in succession to Mr. Gostling. With two dissentients the committee expressed their willingness to purchase the Wheeley's Gardens property for £4,450.

"We ought to rejoice," says the *Sussex Coast Mercury*, "that, divided as we are in our views upon religion, politics, and nearly everything else that admits of a contrariety of opinion, we have not yet discovered any reason why the hand of charity should be stayed when disease and suffering require that it should be put forth."

In connexion with the dreadful murder of Major Hare at Surbiton, and the suicide of the murderer, Gordon Hare, it was stated at the inquest that the latter had lately been in the habit of taking some stimulant supplied by a chemist. It would be easy to make too much of this simple circumstance; nevertheless, it is incumbent upon chemists to recognise the fact that they are empowered to sell articles the frequent use of which may temporarily unbalance the mind. There is quite a possibility that such an aberration as the too frequent use of certain medicinal stimulants would produce, may have turned the scale which resulted in the deplorable tragedy at Surbiton.

The number of inmates of Woodbury Hill Reformatory for Boys at Shelsley, when it was recently inspected by Colonel Sugley, the inspector of reformatory and industrial schools, was 58, and 16 on licence. The premises were in effective order, and the inspector found all homelike and comfortable, but he would gladly see the old dormitory pulled down and rebuilt so as to give more room for the number under detention. The general accommodation is but inferior; at the same time all is kept clean and comfortable and made the most of. The situation is a particularly healthy one, in a remote but beautiful locality. The lads looked healthy and thriving. There had been very little illness; only one boy had died. The boys had been going on pretty steadily. Two absconded, but were recovered. The lads were well behaved and under good influence. As to education, the boys were very willing and tractable; a pleasant set of promising boys. The occupation is chiefly farm and garden labour. The average number maintained was 58; the total cost for 1887 was £969 16*s.* 4*d.* The net cost per head (including industrial profit, £116 5*s.* 11*d.*) £19 8*s.* 9*d.* Of the 56 cases discharged during 1884-5-6 there are doing well, 42; dead, 5; doubtful, 3; convicted of crime, 6.

THE following vacancies are announced this week, the amount of salary in each case being given after the name of the institution :—

**Resident Medical Officers.**

Belgrave Hospital for Children. Resident House Surgeon and Assistant Secretary.—£30 per annum.
Cheltenham General Hospital. Resident Surgeon for the Branch Dispensary.—£180 per annum, with partly furnished house.
Edinburgh City Poorhouse, Craig Lockhart. Resident Medical Officer.—£80 per annum, with board.
General Hospital, Birmingham. Assistant House Surgeon.
General Infirmary, Leeds. Resident Medical Officer and Pathologist —£100 per annum, and board, etc.
Liverpool Northern Hospital. Assistant House Surgeon.—£70 per annum, with board.
Rotherham Hospital.—Board; no salary.
Royal Hospital of Bethlem. Assistant Medical Officer.—£300 per annum, with furnished house.
St. Mark's Hospital for Fistula, etc., City Road, E.C. House Surgeon.—£50 per annum, with board, etc.

**Non-resident Medical Officers.**

King's County Infirmary, Tullamore. Surgeon.—£94 per annum.
National Lying-in Hospital, Dublin. Assistant to the Master.
University of Aberdeen. Chair of Chemistry.
Winchcombe Union. Medical Officer.—£65 per annum, with fees.

A CHARGE has been made by the Chadderton (Lancashire) local authorities that "the Westhulme Hospital has been spreading small-pox in Chadderton and more particularly in Burnley Lane during the present year." To this Dr. Niven, the medical officer of health for Oldham, replies in a lengthy report, the gist of which is that, in Dr. Niven's words, "There is not sufficient reason for a serious charge against Westhulme Hospital; nor is there, in my opinion, sufficient ground even for grave suspicion." This is clearly a case of doctors disagreeing. Which of the disputants is right, who shall say? One thing is noticeable, that Dr. Niven expresses himself with admirable caution, and manages to speak a good deal without saying very much.

THE Duchess of Connaught has attended a St. John Ambulance class for ladies at Poona, and has been awarded a certificate, after passing a very satisfactory examination. Two large classes for men of the 1st Royal Fusiliers and 2nd Durham Light Infantry have also been held by the same lecturer, Dr. Kilkelly, Army Medical Staff, and the certificates, forty-one in number, were presented on the 27th ult. by the Duke of Connaught at the Soldiers' Institute, Poona. His Royal Highness, who was attended by his staff, was received by Major-General Solly-Flood, commanding the division, and the members of the divisional and medical staffs and many officers of the garrison. Having witnessed a very effective ambulance demonstrat ion bythe classes, the Duke presented the certificates, and in a short speech expressed his gratification at being present and at the proficiency displayed.

THE recent agitation about providing nurses for those who cannot pay for the entire services of a trained attendant has brought out the fact that many institutions send out nurses by the day or night when desired. As many families would gladly avail themselves of such aid if they knew where to obtain it, we are pleased to bring them before the notice of our readers. One of these institutions is the Belgravia Institute for Trained Nurses, at 25, Lupus Street, St. George's Square, S.W., of which Mrs. G. L. Bedingfield is superintendent. Besides going out for short periods, the nurses of the Belgravia Institute will attend a case to apply leeches, dress blisters, give enemata, and perform similar services which the patient's friends, however able and willing to do all that is needed in the way of care and attendance, are incompetent to undertake. We are sure that many people now forced to choose between the kind, but untrained, nursing of their relatives and the expense of a nurse's fees and board, would gladly pay the moderate sum charged for such attentions as these.

THE Working Men's Committee in aid of the Royal Hospital for Diseases of the Chest, City Road, have arranged to hold a public meeting in St. Clement's Hall, Lever Street, City Road, on Wednesday, when the chair will be taken by Mr. Nicholson, President of the Mile End New Town Working Men's Society.

AT the recent annual meeting of the Barton-under-Needword Cottage Hospital, a letter was read from Sir Reginald Hardy, expressing the intention formed by himself and his brothers, to set apart a sum of £11,000, the interest of which is to be devoted to the hospital, in compliance with a wish expressed by his father, the late Sir John Hardy, shortly before his death. The hospital goes on increasing its work of modest usefulness, and the care of the hon. secretary, Mr. T. F. Reeve, aided by the secretary of the ladies' committee, Miss F. Lyon, secures that its management shall be satisfactory alike to the trustees and committee, and to the patients treated in it, among whom there was not one death during the year. The report also specially states that Nurse Aldridge, who has charge of the hospital, continues to merit high commendation.

THE Merchant Taylors' Convalescent Home for Ladies at Bognor is conducted on exceptionally generous principles. Not only is residence in the Home for such time as the company's medical officer may think necessary entirely gratuitous, but each patient considered eligible for it receives a free pass by railway from London to Bognor and back. The rules of the institution are by no means irksome, being adapted to the habits and requirements of the class for whom the Home is intended—viz., the wives, widows, or daughters of gentlemen, who, recovering from illness or suffering from the effects of overwork, require rest and sea air to restore them to health, but whose means are insufficient to procure it. Candidates for admission should apply to Mr. F. G. Faithfull, clerk of the company, Merchant Taylors' Hall, Threadneedle Street, E.C., for a preliminary form of application.

AN ornamental tablet has recently been erected at Guy's Hospital to the memory of Dr. Moxon, whose brilliant career as a physician and teacher of medicine ended July 21st, 1886, at the age of 50. The following is the inscription: "In memory of Walter Moxon, M.D., physician to and lecturer on Medicine at Guy's Hospital, whose genius, energy, and personal charm of manner enabled him to obtain a distinguished and unique position in his profession, and endeared him to his colleagues, pupils, and friends, by whom this tablet has been erected, and a gold medal, founded for clinical observation and research, to be awarded triennially by the Royal College of Physicians. He died 21st July, 1886, aged 50 years." The profession of the deceased is allegorically represented by the figures of an aged man, and a mother nursing a sick child. It is designed and executed by Messrs. Barkcutin and Krall, 289, Regent Street.

THE Society for the Prevention of Cruelty to Children, 7, Harpur Street, Bloomsbury, have prepared a Bill, which is directed against the employment, under certain conditions, of children under fourteen years of age. Any child under that age must not be taken out to beg, or to accompany beggars, or to sing or play on any instrument. Another clause prohibits the employment at theatres and places of entertainment of all children under ten years of age Another gives a judge power in case of a conviction, when it is shown that the prisoner was pecuniarily interested in the death of the child, to impose a fine up to a hundred pounds in amount. There is also a valuable provision authorising a magistrate to issue a warrant directing that a cruelly-treated child shall be at once removed to a place of safety. The Bill is backed by Mr. Mundella, Mr. John Morley, Sir Stafford Northcote, Bart., Alderman Sir R. N. Fowler, Bart., Sir H. James, Q.C., Mr. Robert Reid, and Mr. Channing

